# Elastic nailing versus plate and screws fixation for pediatric femoral shaft fractures: a systematic review and meta-analysis of high quality randomized controlled trials

**DOI:** 10.3389/fped.2026.1803368

**Published:** 2026-04-28

**Authors:** Majed N. Alosaimi, Rayan H. Abuhadi, Khaled M. Qanash, Saleh G. Alqadi, Abdullah B. Alsharif, Shoq F. Alghamdi, Jana Y. Aljohani, Lujain K. Anbari, Ahmed H. Kaneetah, Saeed I. Alqahtani

**Affiliations:** 1 King Abdullah International Medical Research Center, Jeddah, Saudi Arabia; 2 College of Medicine, King Saud bin Abdulaziz University for Health Sciences, Jeddah, Saudi Arabia; 3 Department of Surgery, Orthopedic Division, King Abdullah Specialized Children’s Hospital, Ministry of the National Guard-Health Affairs, Jeddah, Saudi Arabia.

**Keywords:** elastic stable intramedullary nailing, meta-analysis, pediatric femoral shaft fractures, plate fixation, systematic review, titaniumelastic nail system

## Abstract

**Introduction:**

Femoral shaft fractures are the most common pediatric femoral injury, accounting for 64%–68% of cases in children under 18 years. While younger children are usually treated non-surgically, older children often require fixation. Elastic stable intramedullary nailing (ESIN) and plating are the two main surgical options, but the optimal method remains debated.

**Methods:**

This systematic review and meta-analysis followed PRISMA guidelines and was registered in PROSPERO. Medline/PubMed, ScienceDirect, Scopus, and Embase were searched for randomized controlled trials (RCTs) comparing ESIN and plating in patients ≤18 years with diaphyseal femoral fractures. Two reviewers independently extracted data, and study quality was assessed using the Cochrane Risk of Bias 2 tool. Meta-analyses were performed in RevMan 5.4.1 using random-effects models with significance set at *P* < 0.05.

**Results:**

Six RCTs including 364 children (182 ESIN, 182 plating) were analyzed. ESIN significantly reduced operative time (MD −36.76 min, *P* = 0.03) and blood loss (MD −94.77 mL, *P* < 0.00001) compared with plating. No significant differences were found in fracture union (MD −1.30 weeks, *P* = 0.16) or hospital stay (MD −3.80 days, *P* = 0.18). ESIN was associated with implant-related irritation, while plating showed higher risks of deep infection and postoperative stiffness.

**Conclusions:**

ESIN offers shorter operative time and less blood loss but presents different complications compared with plating. Larger multicenter RCTs with extended follow-up are needed for definitive guidance.

**Systematic Review Registration:**

https://www.crd.york.ac.uk/PROSPERO/view/CRD420251111355, PROSPERO CRD420251111355.

## 1 Introduction

The femoral shaft occupies a central role in pediatric trauma due to its biomechanical and anatomical characteristics. As the longest and most robust bone of the human skeleton, the femur is essential for weight transmission and locomotor stability, and its disruption carries significant functional and socioeconomic consequences (1). In children, fractures of the diaphysis constitute the most common femoral injury, reflecting both the biomechanical vulnerability of this region and the distinctive injury mechanisms encountered across developmental stages (2). Epidemiological data from large national registries in Scandinavia, North America, and the United Kingdom consistently demonstrate that femoral shaft fractures represent 64%–68% of all pediatric femoral fractures, corresponding to approximately 1%–2% of the total pediatric fracture burden (2–4). Notably, the etiology of these injuries varies by age: low-energy domestic falls are characteristic of early childhood, playground injuries and sports predominate in middle childhood, while high-energy trauma such as motor vehicle collisions emerges as the principal cause during adolescence (5). This age-specific epidemiological pattern underscores the complex interplay between skeletal maturity, activity profile, and environmental risk, and highlights the importance of tailoring management strategies to the child's developmental stage (2).

Fracture stability is a key determinant of management. According to the American Academy of Orthopaedic Surgeons (AAOS), fractures that are anatomically aligned and minimally displaced are considered stable, whereas comminuted, long-oblique, and spiral patterns are classified as unstable (6). Specifically, transverse and short-oblique fractures are considered length-stable, whereas long-oblique and spiral fractures are length-unstable. This distinction has important implications for the choice of fixation (2).

Treatment strategies also vary with age. Infants under 6 months are usually managed with a Pavlik harness, while children between 6 months and 5 years are treated with early spica casting. In children older than 6 years, surgical fixation is generally preferred (4). Operative options include elastic stable intramedullary nailing (ESIN), submuscular plating, and, in selected cases, external fixation—most commonly reserved for open fractures, polytrauma, or damage-control situations. The choice among these techniques depends on patient age and weight, fracture location and stability, associated injuries, and surgeon preference (4, 8).

In school-aged children with closed femoral shaft fractures, both ESIN and plating are widely accepted. ESIN provides a minimally invasive technique (7) that facilitates early mobilization, though its effectiveness decreases in heavier children (>50 kg). Plating, on the other hand, ensures rigid fixation and is particularly advantageous in length-unstable or comminuted fractures. The relative merits of these two methods remain a subject of ongoing debate (9).

A recent systematic review and meta-analysis (2023) comparing ESIN and plating in children aged 6–11 years suggested a lower risk of superficial infection with plating but found no significant differences in malunion, reoperation, or leg-length discrepancy. However, the included studies were heterogeneous in design, often limited by small sample sizes and non-standardized outcome definitions, which lowered the certainty of evidence and limited the strength of clinical recommendations (10). A focused synthesis of high-quality randomized controlled trials is therefore warranted.

This systematic review and meta-analysis of randomized controlled trials (RCTs) aims to compare ESIN and plate fixation in children under 18 years with femoral shaft fractures, evaluating surgical, radiographic, and functional outcomes to provide high-level evidence for guiding best practice in pediatric orthopedic trauma.

## 2 Methods

### 2.1 Search strategy

This systematic review was conducted in accordance with the Preferred Reporting Items for Systematic Reviews and Meta-Analyses (PRISMA) guidelines, and the protocol was registered in PROSPERO. A comprehensive search was performed across PubMed/MEDLINE, ScienceDirect, Scopus, and Embase to identify studies comparing elastic stable intramedullary nailing (ESIN) with plate and screws fixation for pediatric diaphyseal femoral shaft fractures. Search strategies combined free-text terms and controlled vocabulary (e.g., “femoral shaft fracture”, “pediatric”, “elastic nailing”, “plate fixation”) using Boolean operators, tailored to each database. Manual searches of reference lists of included studies were also performed to capture additional eligible articles.

Two reviewers independently screened titles and abstracts, followed by full-text review. Any discrepancies were resolved by discussion or, if necessary, by consultation with a third reviewer.

### 2.2 Eligibility criteria

The inclusion criteria that was used, only RCT's, patients aged ≤18 years who were diagnosed with diaphyseal femoral shaft fractures, and studies comparing elastic nailing with plate and screws fixation.

Exclusion criteria were, studies involving adults or mixed age groups without separate pediatric data, non-randomized studies, and pathologic fractures or fractures related to metabolic bone disease.

### 2.3 Data extraction

The data extraction was done through a full-text assessment using a data collection sheet. Two authors independently performed the data extraction, and any discrepancies was resolved by a third author. Extraction included, study details (author, date of publication, study design, and the country), patient demographics (number of patients, age, gender, BMI, side of fracture, associated injuries, and follow-up period), type of implant, mechanism of injury, fracture level, surgical time, length of stay, fracture type, union time, implant irritation, re-opeining rate, malalignment, leg length discrepancy, peri-oberative and total blood loss, transfusion rate, hemoglobin level pre and post operation, plate size, nail diameter, persistent pain, mortality, surgical complications, healing time, and off-bed loading time.

### 2.4 Quality assessment

The methodological quality of the included RCTs was assessed using the Cochrane Risk of Bias 2 (RoB 2) tool, which evaluates five domains of bias: selection, performance, detection, attrition, and reporting. Each domain was rated as low risk, high risk, or some concerns. Any disagreements were resolved through discussion or by consultation with a third reviewer.

### 2.5 Statistical analysis

The data will be analyzed using RevMan 5.4.1 software. Random effects model was employed to account for variability across studies. *P* value < 0.05 will be considered statistically significant, and a 95% confidence interval will be used to assess the precision of the results. Publication bias will be evaluated through a funnel plot. Moreover, if any confounders are identified, a subgroup analysis will be conducted to further discover their effects on the results. Additionally, we utilized an online calculator (VassarStats) to convert reported values from median and range to mean and standard deviation (11).

### 2.6 Ethical consideration

Ethical approval was not required for this study, as it was based solely on data extracted from previously published literature.

## 3 Results

### 3.1 Study selection

**Figure 1:**
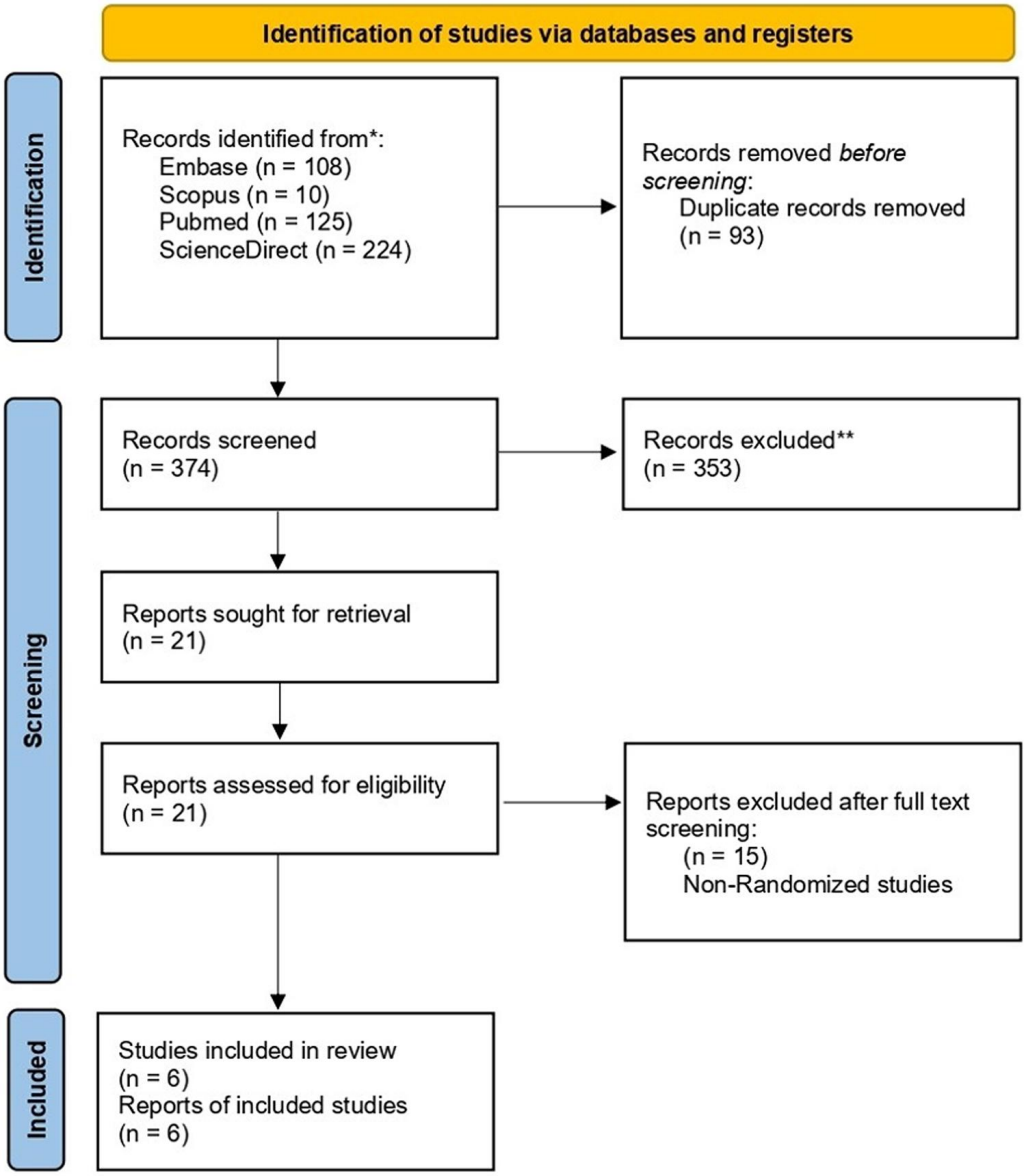
PRISMA flow diagram of study selection.

A systematic search across the selected electronic databases identified 467 records. After removing 93 duplicates, 374 unique studies remained for screening. These underwent a two-step process of title and abstract review, followed by full-text evaluation. At the initial screening stage, 353 articles were excluded based on predefined eligibility criteria, such as inappropriate study design, irrelevant interventions, or unreported outcomes. The remaining 21 full-text articles were assessed in detail. Of these, 15 were excluded due to incomplete data, non-randomized design, absence of a comparator group, or duplicate publication. Studies were eligible for inclusion if they involved patients aged ≤18 years with diaphyseal femoral shaft fractures, treated with either elastic stable intramedullary nailing (TENS) or plate-and-screw fixation, and were designed as randomized controlled trials (RCTs). Studies were excluded if they included adults or mixed age groups without separate pediatric data, were non-randomized, or involved pathologic fractures and fractures related to metabolic bone disease. Ultimately, Six randomized controlled trials (RCTs) met all inclusion criteria and were incorporated into the final systematic review and meta-analysis. The study selection process is illustrated in the PRISMA flow diagram (Figure 1).

### 3.2 Baseline characteristics

A total of six randomized controlled trials comprising 182 pediatric patients in each group (TENS vs. plate-and-screw fixation) with femoral shaft fractures were included (Table 1). The majority of patients were male, with 128 males and 54 females in the TENS group, and 123 males and 59 females in the plate-and-screw group. The mean age across studies ranged from 7.0 ± 2.05 to 10.36 ± 2.19 years in the TENS group and from 6.3 ± 2.21 to 10.2 ± 2.7 years in the plate-and-screw group. The follow-up duration varied among trials, ranging from 6 to 24 months, with most studies reporting outcomes at 3, 6, and 12 months.

**Table 1:** Baseline characteristics of included randomized controlled trials.

Study (year, country)	Patients (TENS)	Patients (plating)	Mean age TENS (±SD)	Mean age plating (±SD)	Sex (M/F) TENS	Sex (M/F) plating	Follow-up
El-Aldly et al. (12), Egypt	25	25	7.96 ± 1.6	8.28 ± 1.6	22/3	18/7	∼12 weeks
Neupane et al. (13), Nepal	10	10	7.0 ± 2.055	6.3 ± 2.214	5/5	9/1	∼12 weeks
James et al. (14), S. India	20	20	9.45 (6–15) (SD 3.1)	10.2 (6–15) (SD 2.7)	13/7	13/7	∼12 weeks
Wang et al. (15), China	60	60	10.36 ± 2.19	6.55 ± 2.06	38/22	36/24	8–14 weeks
Hayat et al. (16), Pakistan	51	51	8.82 ± 1.62	9.27 ± 1.74	41/10	37/14	8–12 weeks
Aldoori et al. (17), Iraq	16	16	7.1 ± 1.4	7.8 ± 1.2	9/7	10/6	10–12 weeks
Total	182	182	—	—	128/54	123/59	—

**Figure 2:**
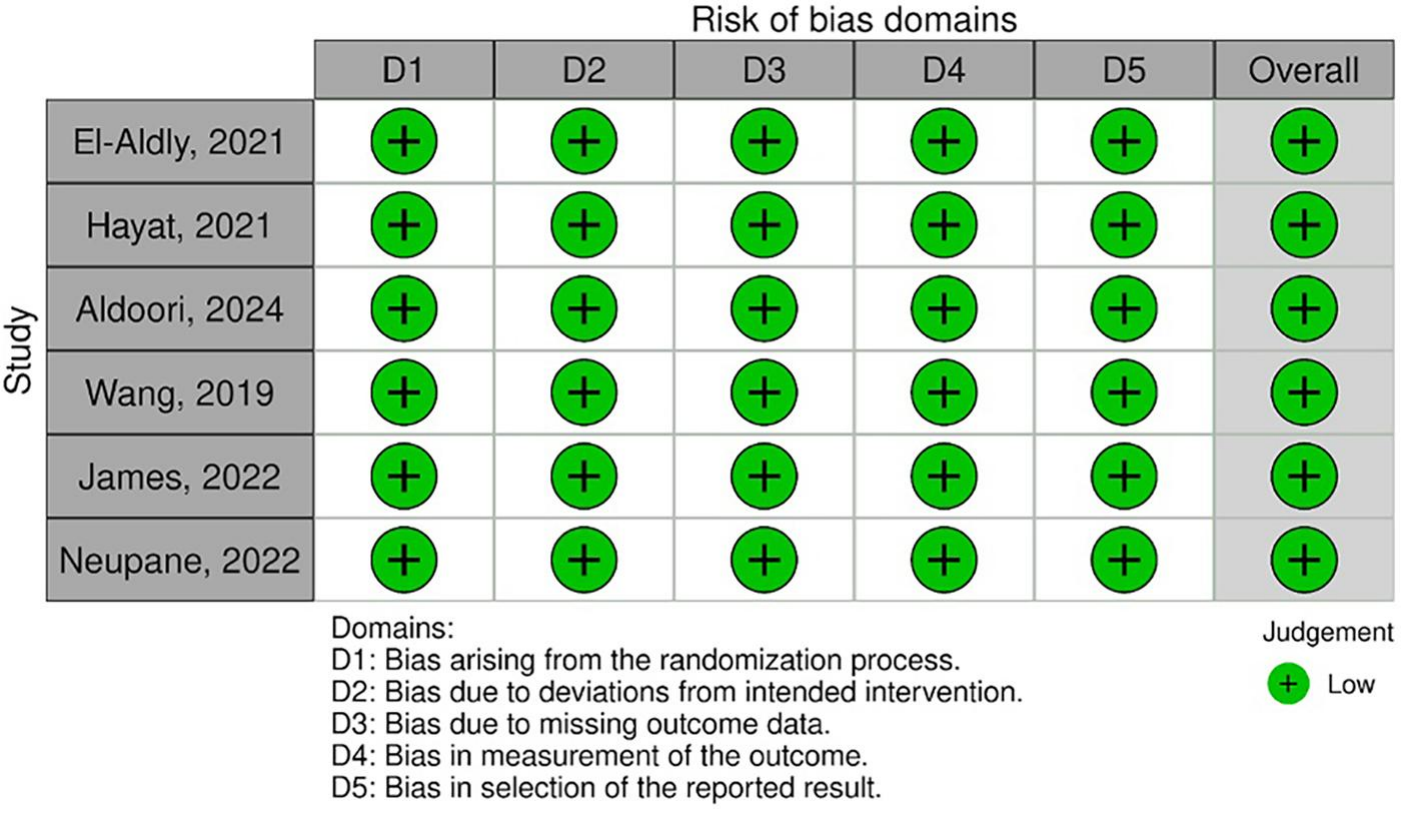
Risk of bias assessment of included randomized controlled trials.

**Figure 3:**
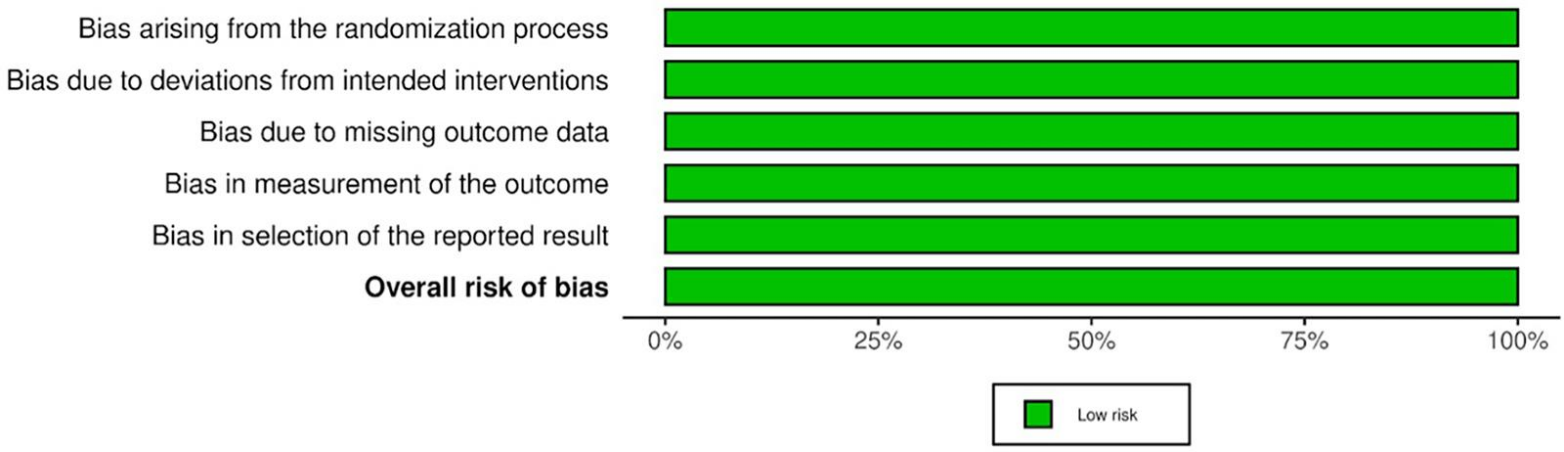
Summary of risk of bias across all included randomized controlled trials.

The risk of bias assessment was conducted using the Cochrane Risk of Bias tool (RoB 2.0). All included studies demonstrated high methodological quality. Across all assessed domains—including the randomization process, deviation from intended intervention, missing outcome data, outcome measurement, and selective reporting—each trial was judged to be at low risk of bias (Figures 2, 3). The included RCTs (12–17) were therefore found to apply sound methodological practices and reported their outcomes transparently. The consistent low risk of bias across all studies enhances confidence in the reliability and validity of the evidence synthesized in this review.

### 3.3 Surgical time

**Figure 4:**
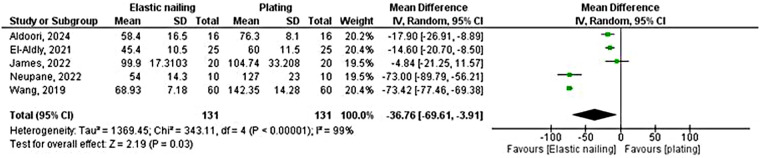
Forest plot comparing surgical time between elastic nailing and plating.

Five randomized controlled trials with a total of 262 patients (131 TENS vs. 131 plating) reported on surgical time (Figure 4). The pooled analysis demonstrated a significantly shorter operative duration in the elastic nailing group compared with plating, with a mean difference of −36.76 min (95% CI −69.61 to −3.91; *P* = 0.03). Heterogeneity among studies was substantial (*I*^2^ = 99%, *P* < 0.00001).

After sensitivity testing, three randomized controlled trials including 61 patients in each group compared surgical time between elastic nailing and plate-and-screw fixation. The pooled analysis demonstrated a significantly shorter operative time with elastic nailing compared to plate-and-screw fixation (MD = –14.70 min, 95% CI −19.53 to −9.87; *P* < 0.00001) (Supplementary Figure S1).

### 3.4 Union time

**Figure 5:**
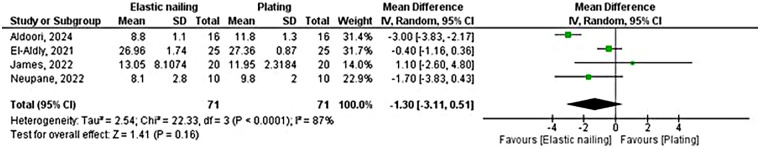
Forest plot comparing fracture union time between elastic nailing and plating.

In terms of union time, four randomized controlled trials with a total of 142 patients compared elastic nailing with plate-and-screw fixation (Figure 5). The pooled analysis showed no significant difference between the two techniques (MD = –1.30 weeks, 95% CI −3.11 to 0.51; *P* = 0.16). The analysis revealed significant heterogeneity (*χ*^2^ = 22.33, df = 3, *P* < 0.0001; *I*^2^ = 87%), suggesting marked differences among study findings.

After sensitivity testing, three randomized controlled trials with a total of 110 patients were included. The findings showed no significant difference in union time between elastic nailing and plating (MD = –0.49 weeks, 95% CI −1.19 to 0.22; *P* = 0.18). The results were consistent across studies, with no evidence of heterogeneity (*χ*^2^ = 2.00, df = 2, *P* = 0.37; *I*^2^ = 0%) (Supplementary Figure S2).

### 3.5 Length of stay

**Figure 6:**
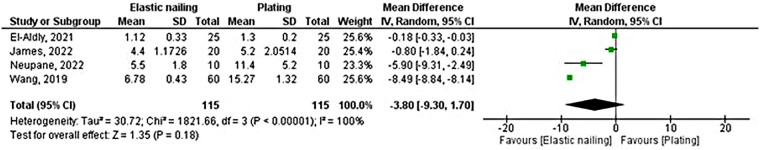
Forest plot comparing hospital length of stay between elastic nailing and plating.

For length of stay, four randomized controlled trials with a total of 230 patients were included (Figure 6). The pooled analysis showed no significant difference between the two groups (MD = –3.80 days, 95% CI −9.30 to 1.70; *P* = 0.18). Marked heterogeneity was observed (*χ*^2^ = 1,821.66, df = 3, *P* < 0.00001; *I*^2^ = 100%), reflecting considerable variability among the study results.

A sensitivity analysis including two randomized controlled trials with 90 patients was conducted to reassess hospital stay duration after excluding outlier studies (Supplementary Figure S3). The pooled result showed no significant difference between elastic nailing and plate-and-screw fixation (MD = –0.27 days, 95% CI −0.70 to 0.16; *P* = 0.22). Heterogeneity was low (*χ*^2^ = 1.35, df = 1, *P* = 0.25; *I*^2^ = 26%), suggesting consistent findings across the included trials.

### 3.6 Peri-operative blood loss

**Figure 7:**
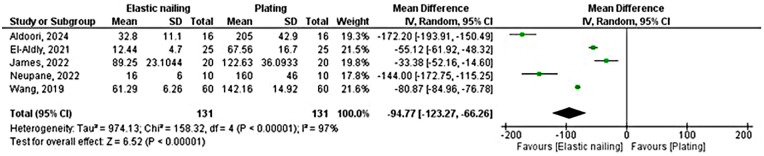
Forest plot comparing peri-operative blood loss between elastic nailing and plating.

Five randomized controlled trials compared peri-operative blood loss in patients undergoing elastic nailing vs. plate-and-screw fixation, with a total of 131 patients in each group (Figure 7). The pooled analysis showed significantly lower blood loss in the elastic nailing group (MD = –94.77 mL, 95% CI −123.27 to −66.26; *P* < 0.00001). However, heterogeneity was very high (*χ*^2^ = 158.32, df = 4, *P* < 0.00001; *I*^2^ = 97%), indicating substantial variation among studies.

After sensitivity testing, two randomized controlled trials with 52 patients were included. They showed significantly reduced peri-operative blood loss in the elastic nailing group compared with plating (MD = –159.74 mL, 95% CI −187.19 to −132.29; *P* < 0.00001). Moderate heterogeneity was present (*χ*^2^ = 2.35, df = 1, *P* = 0.13; I² = 58%), but the direction of effect remained consistent across studies (Supplementary Figure S4).

### 3.7 Complications

Across the six included RCTs (12–17), several postoperative complications were reported for both fixation methods (Table 2). In the TENS group, complications were predominantly implant or soft tissue related. El-Aldly et al. (12) reported four cases of soft tissue irritation and one delayed union, while Neupane et al. (13) documented two implant failures, two prominent painful implants, and one case of genu valgum. James et al. (14) observed two skin infections and one case of ankylosis, and Wang et al. (15) reported three superficial infections (5.9%). Hayat et al. (16) further noted five cases of skin irritation and six cases of hardware discomfort. By contrast, Aldoori et al. (17), assessing plate-and-screw fixation, identified two superficial infections, one deep surgical site infection (1.9%), one prominent painful implant, one case of genu valgum, two cases of ankylosis, and three cases of hardware discomfort. Collectively, these findings suggest that TENS fixation is more frequently associated with implant-related irritation and minor soft tissue complications, whereas plate-and-screw fixation demonstrates a higher risk of deep infection and joint stiffness.

**Table 2:** Postoperative complications in elastic nailing versus plate-and-screw fixation.

Complication	TENS (*n*/%)	Plate-and-screw (*n*/%)
Soft tissue irritation	4 cases	N/A
Delayed union	1 case	N/A
Implant failure	2 cases	N/A
Prominent/painful implant	2 cases	1 case
Genu valgum	1 case	1 case
Skin infection (superficial)	2 cases (+3 cases, 5.9%)	2 cases
Deep surgical site infection	—	1 case (1.9%)
Ankylosis	1 case	2 cases
Hardware discomfort	6 cases	3 cases
Skin irritation	5 cases	—
Total reported complications	Multiple events across studies	Multiple events across studies

## 4 Discussion

This systematic review and meta-analysis compare elastic nailing and plating for treating pediatric femoral shaft fractures. It includes six randomized controlled trials (RCTs) involving a total of 364 pediatric patients. The primary outcomes assessed were hospital length of stay, perioperative blood loss, time to union, surgical duration, and complication rate.

The results suggest that elastic nailing has several advantages over plating, particularly in terms of reduced perioperative blood loss and shorter surgical time. Although time to union and hospital stay did not show statistically significant differences, there was a trend favoring elastic nailing in both outcomes. This can likely be attributed to the less invasive nature of elastic nailing, which requires smaller incisions and involves a simpler insertion technique compared to plating.

Our findings are consistent with previous systematic reviews, such as Liu et al. (18), which reported shorter surgical times and reduced blood loss with ESIN. Similarly, Hu et al. (22) emphasized the higher frequency of implant irritation in ESIN compared with plating, mirroring our conclusions regarding complication profiles. However, unlike some prior reviews that pooled heterogeneous study designs, our restriction to RCTs strengthens the internal validity of these findings. In contrast, a recent meta-analysis of mixed study designs (2023) suggested a slightly lower risk of superficial infection with plating but found no differences in union or reoperation rates. The discrepancy may be explained by differences in study design quality and outcome definitions. Our results, derived exclusively from randomized data, suggest that the risk of deep infection is more concerning with plating, aligning with the biological rationale of larger incisions and greater soft-tissue dissection.

By focusing on randomized controlled trials (RCTs), we ensured the inclusion of high-quality evidence, which minimizes biases common in non-randomized studies, such as selection bias. Additionally, we followed a rigorous methodology that included a comprehensive search strategy using MeSH terms across four databases and adherence to PRISMA guidelines (19). The high heterogeneity observed in several outcomes may be explained by differences across the included trials in terms of patient demographics, fracture characteristics, surgical techniques, and perioperative protocols. Variations in follow-up duration and outcome reporting methods may also have contributed to statistical heterogeneity. Because only six randomized controlled trials were included and several potentially relevant variables were inconsistently reported, formal subgroup analyses or meta-regression were not feasible. Our review was registered in PROSPERO, further enhancing the methodological rigor and credibility of our findings.

However, a limitation of this review is the relatively small sample size, which may limit the external applicability of the results. Additionally, the inclusion of only four databases may have resulted in missing unpublished studies or those available in other databases, which could introduce publication bias (20). Moreover, the reliance on short-term follow-up data restricts the completeness of the findings, suggesting the need for future studies with longer follow-up periods to better understand long-term outcomes. Also, some missing data from certain studies can affect the generalizability of the results. Another limitation is the inability to formally assess publication bias. Because fewer than ten studies were included, funnel plot analysis would have limited reliability and may lead to misleading conclusions. Furthermore, functional outcomes were inconsistently reported across the included trials, which prevented quantitative analysis of patient-centered functional recovery.

Elastic nailing appears to be the preferred choice over plating, especially for closed fractures, due to its trend toward faster union times, which is particularly beneficial for pediatric patients who require quicker returns to normal activities. Elastic nailing also involves shorter surgical durations, benefiting both the patient and the surgeon. It results in less perioperative blood loss, helping to maintain hemoglobin levels and reducing the need for blood transfusions, which in turn conserves hospital resources. Additionally, the shorter length of hospital stay associated with elastic nailing leads to higher patient satisfaction, reduced risk of hospital-acquired infections, and lower healthcare costs (21). Despite these promising findings, further research with larger sample sizes and longer follow-up periods is necessary to provide more precise and reliable results.

## 5 Conclusion

This meta-analysis of RCTs provides high-level evidence that elastic stable intramedullary nailing offers significant perioperative advantages over plating, with shorter surgical duration and reduced perioperative blood loss, while achieving comparable union times and hospital stays. Although ESIN is associated with more frequent minor implant-related complications, plating carries a higher risk of deep infection and joint stiffness.

Taken together, these findings suggest that elastic nailing should be considered the first-line fixation method for length-stable pediatric femoral shaft fractures, while plating remains a valuable option for unstable or comminuted fracture patterns. Future large-scale, multicenter RCTs with standardized outcome reporting and long-term follow-up are needed to refine patient selection criteria and optimize outcomes in this important pediatric population.

## Data Availability

The datasets presented in this study can be found in online repositories. The names of the repository/repositories and accession number(s) can be found in the article/supplementary material.
