# Supplementary material for: Elastic nailing versus plate and screws fixation for pediatric femoral shaft fractures: a systematic review and meta-analysis of high quality randomized controlled trials
Source: Front Pediatr. 2026 Apr 28;14:1803368. doi: 10.3389/fped.2026.1803368 (PMC13161135; doi:10.3389/fped.2026.1803368)
Supplement: Supplementary file 1 [file Supplementaryfile1.docx]

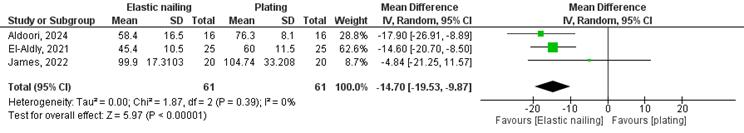


**Supplementary Figure 1.** Sensitivity analysis of surgical time between elastic nailing and plating.


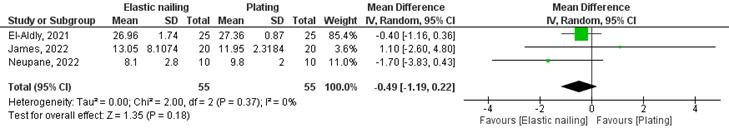


**Supplementary Figure 2.** Sensitivity analysis of fracture union time between elastic nailing and plating.


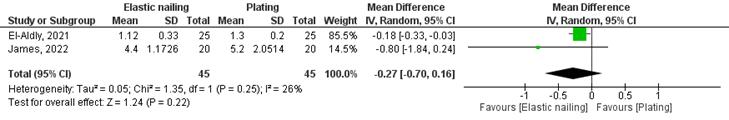


**Supplementary Figure 3.** Sensitivity analysis of hospital length of stay between elastic nailing and plating.


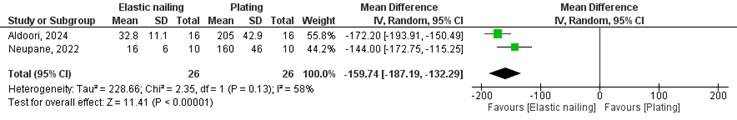


**Supplementary Figure 4.** Sensitivity analysis of peri-operative blood loss between elastic nailing and plating.
